# A Time-Series-Based Feature Extraction Approach for Prediction of Protein Structural Class

**DOI:** 10.1155/2008/235451

**Published:** 2008-03-26

**Authors:** Ravi Gupta, Ankush Mittal, Kuldip Singh

**Affiliations:** 1 Department of Electronics and Computer Engineering, Indian Institute of Technology Roorkee, Roorkee 247 667, Uttarakhand, India; 2 Information Science Division, AU-KBC Research Centre, Anna University, MIT Campus, Chennai 600 044, India

## Abstract

This paper presents a novel feature vector based on physicochemical property of amino acids for prediction protein structural classes. The proposed method is divided into three different stages. First, a discrete time series representation to protein sequences using physicochemical scale is provided. Later on, a wavelet-based time-series technique is proposed for extracting features from mapped amino acid sequence and a fixed length feature vector for classification is constructed. The proposed feature space summarizes the variance information of ten different biological properties of amino acids. Finally, an optimized support vector machine model is constructed for prediction of each protein structural class. The proposed approach is evaluated using leave-one-out cross-validation tests on two standard datasets. Comparison of our result with existing approaches shows that overall accuracy achieved by our approach is better than exiting methods.

## 1. Introduction

Determination of protein structure from its primary sequence is an active area of research in bioinformatics. The knowledge of protein structures plays an important role in understanding their functions. Understanding the rules relating the amino acid sequence to the three-dimensional structure of the protein is one of the major goals of contemporary molecular biology. However, despite more than three decades of both experimental and theoretical efforts prediction of protein structure still remains one of the most difficult issues.

The concept of protein structural classes was originally introduced by Levitt and Chothia [1] based on a visual inspection of polypeptide chain topologies in a dataset of 31 globular proteins. A protein (domain) is usually classified into one of the following four structural classes: , , , and . Structural class categorizes various proteins into groups that share similarities in the local folding patterns. The  and  classes represent structures that consist of mainly *α*-helices and *β*-strands, respectively. The  and  classes contain both *α*-helices and *β*-sheets where the  class includes mainly parallel *α*-helices and *β*-strands and  class includes those in which *α*-helices and *β*-strands are largely segregated. Prediction of structural classes is based on identifying these folding patterns based on thousands of already categorized proteins, and applying these patterns to unknown structures but known amino acid sequences. Structural Classification of Proteins (SCOP) [2] is one of the most accurate classifications of protein structural classes and has been constructed by visual inspection and comparison of structures by experts.

In the past two decades several computational techniques for prediction of protein structural classes have been proposed. Prediction is usually a two-step process. In the first step a fixed length feature vector is formed from protein sequences which are of different length. The second step involves a classification algorithm. Klein and Delisi [3] proposed a method for predicting protein structural classes from amino acid sequence. Later on, Klein [4] presented a discriminant analysis based technique for this problem. Zhou et al. [5] in 1992 proposed a weighting method to predict protein structural class from amino acids. A maximum component coefficient method was proposed by Zhang and Chou [6]. A neural network based approach [7] for protein structural classes was also developed using six hydrophobic amino acid patterns together with amino acid composition. A new algorithm that takes into account the coupling effect among different amino acid components of a protein by a covariance matrix is proposed in [8]. In [9], Chou and Zhang introduced Mahalanobis distance to reflect the coupling effect among different amino acids components, improving the accuracy of the current problem. A support vector machine (SVM) method using amino acid composition features for prediction of protein structural class was presented by Cai et al. [10] in 2001 and is one of the most accurate methods for classification. A supervised fuzzy clustering approach based on amino acid composition features was introduced by Shen et al. [11]. A combined approach, LogitBoost, was proposed by Feng et al. [12]. It combines many weak classifiers together to build a stronger classifier. In 2006, Cao et al. [13] proposed a rough set algorithm based on amino acid compositions and 8 physicochemical properties data.

In this paper, a three step procedure is proposed for prediction of protein structural class. The main contribution of this paper is in providing a novel feature vector which is obtained by applying a wavelet-based time-series analysis approach. The proposed feature extraction from protein sequence is inspired from the work of Vannucci and Lio [14] on transmembrane proteins. The fixed length feature vector for classification proposed is derived from ten physicochemical properties of protein sequences. The physicochemical properties are used to convert the protein sequences from symbolic domain to numeric domain and to derive a time series representation for protein sequences. Features are extracted by applying a wavelet-based analysis technique for time series data on mapped protein sequences. The feature vector summarizes the variation of physicochemical properties in the protein sequence. Finally, a support vector machine is trained using the novel feature vector and the parameters are optimized for generating accurate model (providing highest prediction accuracy).

Leave-one-out cross-validation also called jackknife test was performed on the datasets that were constructed by Zhou [15] from SCOP. The datasets were also used by Cai et al. [10], Cao et al. [13] for their experiments. An overall accuracy of 82.97% and 93.94% was achieved for 277 domains and 498 domains datasets, respectively, using the proposed approach.

The paper is organized as follows. In Section 2, we describe the steps followed for extracting wavelet variance features from protein sequences. A brief introduction to support vector machine (SVM) is also provided in this section. Section 3 provides the experiment results obtained for datasets of structural protein sequences. Conclusion follows in Section 4.

## 2. Method

The proposed approach for identification of structural classes of proteins is divided into three different stages: amino acid mapping, feature extraction, and classification. In the first stage the protein sequences are mapped to various physicochemical scales as provided in the literature. After this mapping procedure the protein sequences become discrete time series data. The second stage involves construction of fixed length feature vector for classification. The feature vector is generated by combining wavelet variance [16] features extracted from different physicochemical scales used for mapping stage. Finally, an SVM-based classification is performed based on the novel extracted features to identify the structural class of a protein sequence.

### 2.1.Amino Acid Mapping

In this stage, ten different physicochemical amino acid properties were used. The first is the average flexibility indices provided by Bhaskaran and Ponnuswamy [17]. The second is the normalized hydrophobicity scales provided by Cid et al. [18]. The third is the transfer free energy given by M. Charton and B. I. Charton [19] and cited by Simon [20]. The fourth is the residue accessible surface area in folded protein provided by Chothia [21]. The fifth is the relative mutability obtained by multiplying the number of observed mutations by the frequency of occurrence of the individual amino acids and is provided by Dayhoff et al. [22]. The sixth is the isoelectric point provided by Zimmerman et al. [23]. The seventh is the polarity of amino acids provided by Grantham [24]. The eight is the volume of amino acid provided by Fauchere et al. [25]. The ninth is the composition of the amino acids provided by Grantham [24]. The tenth is the molecular weight of the amino acids given by Fasman [26]. The numerical indices representing physicochemical property of amino acids were downloaded from http://www.genome.jp/dbget.

### 2.2. Feature Construction

The representation of a protein sequence by a fixed length feature vector is one of the primary tasks of any protein classification technique. In this section, we present a wavelet-based time-series approach for constructing feature vector. Wavelet transform is a technique that decomposes a signal into several groups (vectors) of coefficients. Different coefficient vectors contain information about characteristics of the sequence at different scales. The proposed feature vector contains information about the variability of ten physiochemical properties of protein sequences over different scales. The variability of physiochemical properties is represented in terms of wavelet variance [16].

In the present work, a variation of the orthonormal discrete wavelet transform (DWT) [27,28], called the maximal overlap DWT (MODWT) [29] is applied for feature extraction. In past, MODWT has been applied for analysis of atmospheric data [30] and economic time series data [31,32]. The MODWT is a highly redundant and nonorthogonal transform. The MODWT was selected over DWT because it can handle any sample size *N*, while *J*th order DWT restricts the sample size to multiple of . The property is very useful for analysis of protein sequences, as the length of the sequences is not a multiple of . In addition, MODWT yields an estimator of the variance of the wavelet coefficients that is statistically more efficient than the corresponding estimator based on the DWT.

Let **P** be an *N*-dimensional column vector containing the mapped protein sequence series  where *N* is the length of the protein sequence. It is assumed that  was collected at time *t*Δ*t*, where Δ*t* is the time interval between consecutive observation (in the present case Δ*t* is equal to 1 amino acid). The MODWT of **P** for maximum level *J* is given by(1)

where **Q** is a column vector of length , and  is an  real-valued nonorthogonal matrix. The vector of MODWT coefficients given in (1) may be decomposed into  vectors:(2)

where  (where ) and  are column vectors of length *N*. The vector  contains the MODWT wavelet coefficients associated with change in **P** on scale of length , while  is a vector containing the MODWT scaling coefficients associated variation at scales of length  and higher. In addition to MODWT coefficients, the matrix  can be decomposed into  submatrices, each of them  and is given by(3)

Instead of using the wavelet and scaling filters, the MODWT utilizes the rescaled filters, that is,  and  (where, ). The terms  and  are wavelet and scaling filters, respectively. The wavelet filter approximates high-pass filter, and the scaling filter approximates low pass filter. Details regarding wavelet and scaling filters can be found in [29]. The  dimensional submatrix  is constructed by circularly shifting the rescaled wavelet filter  by integer units to the right so that(4)

Similarly,  can be obtained. The MODWT is an energy-preserving transform [29,33] and is given as(5)

The sample variance (empirical power) of **P** is decomposed into pieces that are associated with scales (6)

where  is the sample variance of **P**, and  is its mean. The term  represents the contribution to the sample variance of **P** due to change at scale . For example, the average flexibility indices property of a protein sequence in terms of wavelet variance vector is given as follows:(7)

where *J* is the maximum level of decomposition of the time series data, that is, protein sequence. Similarly, wavelet variance vectors for hydrophobicity, transfer free energy, residue accessible surface area, relative mutability, isoelectric point, polarity, volume, composition, and molecular weight are calculated and are represented by  respectively. The feature vector  is constructed by concatenating all seven wavelet variance vectors and is given as follows:(8)

The physiochemical variation of a protein sequence is summarized in the proposed feature vector. The dimension of  is equal to  and is dependent on the number of levels (*J*) to which the time series data (i.e., protein sequence) has to be decomposed. The value of *J* is further dependent on the length of time series data (i.e, protein sequence length) and , where *N* is the number of observation points in the time series or the length of protein. As most of the protein sequences taken up for the experiment have length greater than 32, we have selected . In this study, Daubechies [27] wavelet has been used for analysis.

### 2.3. Classification

The SVM was proposed by Cortes and Vapnik [34] as a very effective technique for pattern classification. SVM is based on the principle of structural risk minimization (SRM), which bounds the generalization error to the sum of training set error and a term depending on the Vapnik-Chervonenkis dimension [34] of the learning machine. The SVM induction principle minimizes an upper bound on the error rate of a learning machine on test data (i.e., generalization error), rather than minimizing the training error itself which is used in empirical risk minimization. This helps them to generalize well on the unseen data.

An open-source SVM implementation called LIBSVM [35] was used for classification. It provides various kernel types: radial basis function (RBF), linear, polynomial and sigmoid. Experiments were conducted using different kernels; however the RBF was selected because of its superior performance for the current work. Further, for finding the optimum values of parameters  for RBF kernel, LIBSVM provides an automatic grid search technique using cross-validation. Basically various pairs of  are tried and the one that provides best cross-validation accuracy is selected.

## 3. Experimental Results

To evaluate the performance of our approach two datasets of protein sequences constructed by Zhou [15] are used. The first dataset consists of 277 domains, of which 70 are  domains, 61  domains, 81 are  domains, and 65 are  domains. The second dataset consists of 498 domains, of which 107 are  domains, 126  domains, 136 are  domains, and 129 are  domains. The datasets were preprocessed before using for the experiment. The protein sequences having length less than 32 amino acids (as , where *J* is the maximum level of decomposition for wavelet transform) were removed from the dataset. The number of protein sequences obtained after preprocessing both datasets is provided in Table 1.

**Table 1:** Dataset for the current study.

					Total
Dataset1	69	61	81	65	276
Dataset2	105	126	135	129	495

The performance of the SVM classifier is measured using leave-one-out cross-validation (LOOCV) technique. LOOCV is *n*-fold cross-validation, where "*n*" is the number of instances in the datatset. Each instance in turn is left out, and the learning method is trained on all the remaining instances. It is judged by its correctness on the remaining instances-one or zero success or failure, respectively. The results of all "*n*" judgments, one for each member of the dataset, are averaged, and that average represents the final error estimate.

The classification of a protein sequence into one of the four structural classes is a multi-class classification problem. For identifying four different structural classes one-versus-others approach was followed. Four different SVMs were constructed, each specific to one class. The *k*th SVM was trained with all the samples of the *k*th class with positive labels and samples of remaining classes with negative labels. For example (Table 2, column 1 and Table 3, column 1), the SVMs for  domains protein sequences are positive labeled where as  domains,  domains, and  domains protein sequences are negative labeled. The experimental results obtained from the four SVMs for dataset1 and dataset2 are presented in Tables 2 and 3, respectively. The optimal SVM parameters obtained for the experiments are also provided in Tables 2 and 3. The accuracies for the current problem were calculated by applying the standard definition provided by previous work for multiclass protein sequence classification problem using SVM [36–38]. The prediction accuracy of the structural classes and overall prediction accuracy are given by(9)

**Table 2:** Experimental result of one-versus-others test on dataset1 evaluated using LOOCV.

				
True positive (TP)	60	54	72	43
False negative (FN)	9	7	9	22
True negative (TN)	199	208	191	209
False positive (FP)	8	7	4	2
in %	93.84%	94.93%	95.29%	91.67%
Area under curve (AUC)	0.947	0.970	0.986	0
Optimal SVM parameters				
				

**Table 3:** Experimental result of one-versus-others test on dataset2 evaluated using LOOCV.

				
True positive (TP)	98	119	131	117
False negative (FN)	7	7	4	12
True negative (TN)	387	367	352	362
False positive (FP)	3	2	8	4
in %	97.98%	98.18%	97.58%	96.77%
Area under curve (AUC)	0.990	0.994	0.992	0.983
Optimal SVM parameters				
				

where *M* is the total number of protein sequences,  is the number of protein sequences of class "*k*,"  is the number of correctly predicted protein sequences of class "*k*." The accuracy of each class and overall accuracy for datset1 and dataset2 calculated using (9) are shown in Table 4. The overall accuracy obtained by our approach for dataset1 and dataset2 is 82.97% and 93.94% respectively. The overall performance of our approach is better than existing techniques. Further, the receiver operating characteristic (ROC) curve and area under curve (AUC) for the proposed protein structural classification task were also calculated. An ROC curve is a plot of true positive rate as the ordinate versus the false positive rate as the abscissa; for a classifier, it is obtained by continuously varying the threshold associated with the decision function [39]. The ROC and AUC obtained for the one-versus-other experiment of dataset1 and dataset2 are presented in Figures 1 and 2, respectively. The ROC curve shown in Figure 1(a) is obtained when  domains protein sequences in dataset1 are positive labeled, where as  domains,  domains, and  domains protein sequences in dataset1 are negative labeled. Similarly, ROC curve for other classifications is also obtained.

**Table 4:** Comparison of Leave-one-out cross-validation accuracy obtained for protein structural classification problem on the two datasets by our approach and existing approaches.

Dataset	Method	Prediction accuracy for each structural class (%)				Overall accuracy (%)
						
Dataset1	Our approach	86.96	88.52	88.89	66.15	82.97
	Component coupled [ 6]	84.3	82.0	81.5	67.7	79.1
	Neural network [ 7]	68.6	85.2	86.4	56.9	74.7
	SVM [ 10]	74.3	82.0	87.7	72.3	79.4
	Rough sets [ 13]	77.1	77.0	93.8	66.2	79.4
Dataset2	Our approach	**93.33**	**94.44**	**97.04**	**90.7**	**93.94**
	Component coupled [ 6]	93.5	88.9	90.4	84.5	89.2
	Neural network [ 7]	86.0	96.0	88.2	86.0	89.2
	SVM [ 10]	88.8	95.2	96.3	91.5	93.2
	Rough sets [ 13]	87.9	91.3	97.1	86.0	90.8

**Figure 1:**
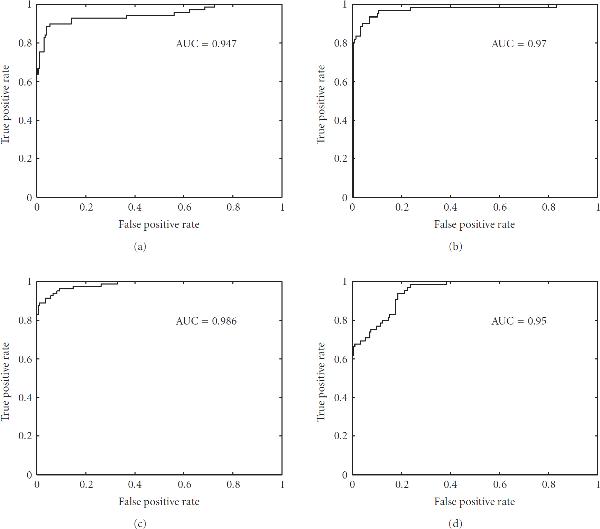
**The ROC curve for identification of four structural classes of dataset1,  domains (a),  domains (b),  domains (c), and  domains (d)**.

**Figure 2:**
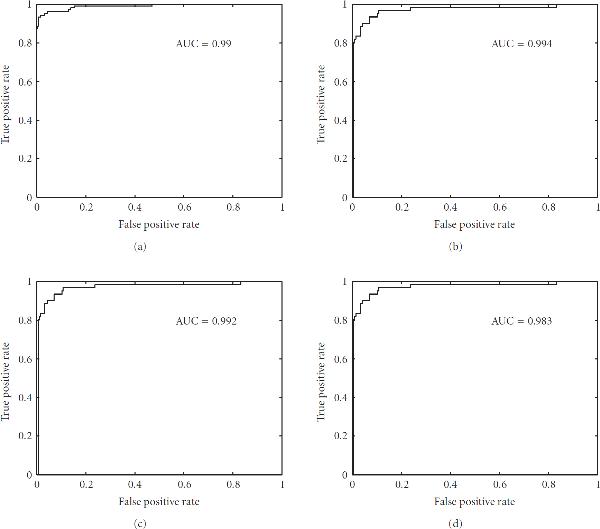
**The ROC curve for identification of four structural classes of dataset2,  domains (a),  domains (b),  domains (c), and  domains (d)**.

## 4. Conclusion

In this work, we have presented a novel wavelet variance based feature vector for prediction of protein structural class. The aim of this research is to provide a new and complementary set of features for the current problem. Based on pattern recognition framework, the proposed approach is divided into three different tasks: amino acid mapping, feature construction, and classification. The feature vector summarizes the variation of ten different physicochemical properties of amino acids. The feature extraction technique is based on wavelet based time series analysis. Experiments were performed on two standard datasets (constructed by Zhou [15]). The result of LOOCV test shows that the proposed method achieves accuracy better than existing methods. The proposed approach can also be applied for identification of membrane protein type, enzyme family classification, and many others.
